# Reconciling the influence of task-set switching and motor inhibition processes on stop signal after-effects

**DOI:** 10.3389/fpsyg.2013.00649

**Published:** 2013-09-24

**Authors:** Joaquin A. Anguera, Kyle Lyman, Theodore P. Zanto, Jacob Bollinger, Adam Gazzaley

**Affiliations:** ^1^Departments of Neurology, Physiology and Psychiatry, Center for Integrative Neurosciences, University of California San FranciscoSan Francisco, CA, USA; ^2^Feinberg School of Medicine, Medical Scientist Training Program, Northwestern UniversityChicago, IL, USA

**Keywords:** motor inhibition, EEG, stop signal, after-effects, ERSP

## Abstract

Executive response functions can be affected by preceding events, even if they are no longer associated with the current task at hand. For example, studies utilizing the stop signal task have reported slower response times to “GO” stimuli when the preceding trial involved the presentation of a “STOP” signal. However, the neural mechanisms that underlie this behavioral after-effect are unclear. To address this, behavioral and electroencephalography (EEG) measures were examined in 18 young adults (18–30 years) on “GO” trials following a previously “Successful Inhibition” trial (pSI), a previously “Failed Inhibition” trial (pFI), and a previous “GO” trial (pGO). Like previous research, slower response times were observed during both pSI and pFI trials (i.e., “GO” trials that were preceded by a successful and unsuccessful inhibition trial, respectively) compared to pGO trials (i.e., “GO” trials that were preceded by another “GO” trial). Interestingly, response time slowing was greater during pSI trials compared to pFI trials, suggesting executive control is influenced by both task set switching and persisting motor inhibition processes. Follow-up behavioral analyses indicated that these effects resulted from between-trial control adjustments rather than repetition priming effects. Analyses of inter-electrode coherence (IEC) and inter-trial coherence (ITC) indicated that both pSI and pFI trials showed greater phase synchrony during the inter-trial interval compared to pGO trials. Unlike the IEC findings, differential ITC was present within the beta and alpha frequency bands in line with the observed behavior (pSI > pFI > pGO), suggestive of more consistent phase synchrony involving motor inhibition processes during the ITI at a regional level. These findings suggest that between-trial control adjustments involved with task-set switching and motor inhibition processes influence subsequent performance, providing new insights into the dynamic nature of executive control.

## Introduction

The act of attempting to inhibit an executed response is one of the best characterized examples of cognitive control. In recent years, response inhibition has been extensively studied through the use of the stop signal paradigm (Logan and Cowan, 1984; Verbruggen and Logan, 2009), with the inhibition process modeled as a horse race between “GO” and “STOP” processes (Logan and Cowan, 1984). This model suggests that the probability of a successful inhibition (SI) depends on the outcome of a race between two independently operating processes (“GO” and “STOP”). While this model describes performance on a given trial, it does not consider how these “GO” and “STOP” processes affect performance on the next trial. Several stop signal studies have shown that response time (RT) to a “GO” signal on trial n is slower when the immediately preceding trial (*n* − 1) was a “STOP” trial vs. a “GO” trial (Rieger and Gauggel, 1999; Verbruggen et al., 2005b; Li et al., 2008; Verbruggen and Logan, 2008). Interestingly, “STOP” trials have only two outcomes, SI or failed inhibition (FI) of the motor response, and RTs during “GO” trials are slowed regardless of whether it follows successful or FI trials. To assess the neural mechanisms underlying these stop signal after-effects, the present study looked to characterize specific neural processes engaged on a “GO” trial when following a trial that contained a previous “Successful Inhibition” (pSI), a previous “Failed Inhibition” (pFI), or a previous “GO” trial (pGO).

Behavioral after-effects are not specific to the stop signal task, as post-error slowing (Rabbitt and Rodgers, 1977) and negative priming (Neill et al., 1990, 1992; Tipper, 2001) studies have regularly reported a similar increase in RTs on subsequent trials. These types of effects have been explained by several different hypotheses of behavior involving task switching (Mayr and Kliegl, 2000; Schneider and Logan, 2005; Kray, 2006), although one is especially relevant to the present investigation: negative priming manifested through the persistence of motor inhibition processes (Kramer et al., 1992; Rieger and Gauggel, 1999). This type of behavior is considered to be indicative of *between-trial control adjustments* (Rieger and Gauggel, 1999), a perspective that is comparable to Allport et al. (1994) “task-set inertia” hypothesis that suggests task features (stimulus-based, and not motor-related) on trial “*n* − 1” can interfere with processing on trial “*n*” when the task requirements change. Thus, responding to a “GO” signal on trial “*n*” requires changing from a “STOP” associated state if the preceding trial contained a “STOP” signal.

However, the between-trial control interpretation has been challenged by evidence suggesting that after-effects following SI performance are actually a reflection of a *repetition-priming effect* (Verbruggen et al., 2008). These researchers examined the directionality of the “GO” signal on a pSI trial vs. the direction on trial *n*, and reported that *only* for trials where the directionality of the “GO” signal repeated were these post-SI “GO” trials slower than repeating “GO” trials (pGO; Verbruggen et al., 2008). Alternatively, when the direction was different, no difference was observed between these trial types, which these authors interpreted as evidence for repetition-priming effects. This finding was consistent regardless of stimulus, category, or even during a selective stop signal task (Verbruggen et al., 2008), with subsequent work demonstrating short-term RT adjustments after unsuccessful stopping and long-term after effects persisting even 20 trials after a SI (Verbruggen and Logan, 2008). However, a more complete understanding of these two positions (repetition-priming effect vs. between-trial control adjustments) may be better understood by corroborating these behavioral effects with the underlying neural processes.

EEG studies of the stop signal task have regularly characterized inhibition-related neural activity using event-related potentials (ERPs; Pliszka et al., 2000; Kok et al., 2004; Ramautar et al., 2004; Schmajuk et al., 2006). These ERPs have characterized the neural activity immediately following a “STOP” event, which does not facilitate the present goal of explaining the effect seen on subsequent “GO” trials. Upton and colleagues (Upton et al., 2010) recently examined N2 and P300 effects on these subsequent “GO” trials, reporting conditional differences that reflect memory retrieval processes with respect to negative priming. However, the act of inhibiting an executed response involves a host of neural regions whose activity is not always best examined post-stimulus, especially considering that these after-effects are influenced by processes occurring during the preceding inter-trial interval (ITI). Indeed, there is a rich literature describing the involvement of different regions such as the right inferior frontal gyrus (rIFG), the medial frontal cortex, and primary motor cortex during stop signal inhibition (Braver et al., 2001; Aron et al., 2007; Jahfari et al., 2010). With respect to stop-signal after effects, activity at any of these regions may be contributing to the reported behavioral effect. Thus, an analysis that facilitates the examination of neural activity at each of these regions prior to the subsequent “GO” stimulus onset may provide a deeper understanding of these after-effects.

One such approach involves the use of frequency based analyses such as coherence (Roach and Mathalon, 2008), as this approach has been shown to be a powerful way of interrogating markers of cognitive control in a spontaneous EEG spectrum (Makeig, 1993; Neuper and Klimesch, 2006). There are several theories postulating that goal-directed behaviors are supported by local synchronization of neural oscillations within specific cortical areas, with this activity integrating spatially distant brain regions into a unified functional network (Tononi and Edelman, 1998; Varela et al., 2001). The examination of single-trial EEG dynamics across theta (4–7 Hz), alpha (8–12 Hz), and beta (15–30 Hz) frequency bands using inter-trial coherence [ITC; a measure of consistency across trials (cf. Makeig et al., 2002)] has been useful in further characterizing activity associated with voluntary response inhibition (Yamanaka and Yamamoto, 2010; Müller and Anokhin, 2012). Similarly, inter-electrode coherence (IEC; a similar measure of consistency between electrodes across trials) has also been used to characterize motor inhibition-related activity from a large-scale network perspective across different frequencies (Shibata et al., 1998; Serrien et al., 2005; Gladwin et al., 2006; Moore et al., 2008; Tallet et al., 2009; Brier et al., 2010; Yamanaka and Yamamoto, 2010; Liang et al., 2012). Specific to interrogating stop signal after-effects, the use of ITC and IEC to examine the temporal and spatial synchronization is theoretically ideal for interrogating motor inhibition processes before (and after) these subsequent “GO” stimuli at different electrodes/regions.

Either IEC or ITC associated with prefrontal, pre-motor, or primary motor areas may reflect the observed stop-signal after effects. However, it is unclear *when* their potential influence would be most apparent, or *how long* this effect would persist: just prior to the subsequent “GO” stimulus, persisting through stimulus onset, or lasting all the way through the subsequent “GO” response itself. Here we hypothesized that both temporal and spatial phase synchrony would increase as greater cognitive control is called for (i.e., following a “STOP” trial), with a conditional change in each type of coherence being the greatest for pSI trials, followed by pFI trials and then pGO trials during the ITI at the electrodes nearest to the aforementioned regions associated with motoric inhibition. We anticipated that this approach would inform these previous behavioral (and more recent ERP) findings by highlighting how well-characterized measures of inhibitory activity are influencing these after-effects that have been attributed to task-switching processes. Thus, the neural signatures underlying these after-effects may provide a deeper understanding of how these potential explanations contribute to the observed behavior in a temporal and regional specific manner.

## Methods

### Participants

Twenty-one healthy young individuals (mean age: 23.5 years; range 18–30 years; 10 males) were recruited from the San Francisco community. These individuals signed a UCSF approved consent form in order to participate in the study and were paid $15/ h for their time. All participants were screened to ensure that they were healthy, had normal to corrected vision and were right handed. EEG data for 3 participants was corrupted during data acquisition, leaving 18 (9 male) participants.

### Experimental procedures

**Figure 1:**
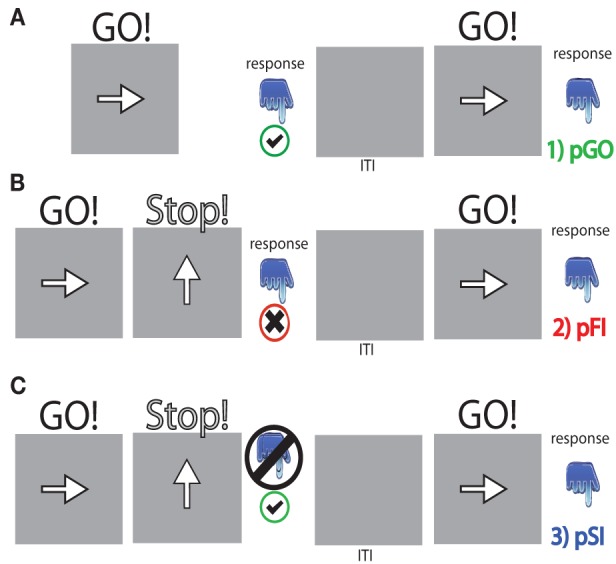
**Task schematic for each trial type. (A)** pGO = a “GO” trial following a “GO” trial, **(B)** pFI = a “GO” trial following a failed inhibition (FI) trial, **(C)** pSI = a “GO” trial following a successful inhibition (SI) trial. “GO” stimuli were presented for [1000 ms—the stop signal delay] calculated for each “STOP” signal event. The inter-trial interval (ITI) was between 1.6 and 1.9 s in length.

The stop signal paradigm consisted of “GO” and “STOP” trials, with each “GO” trial having a left- or right-pointing arrow (the “GO” stimulus) displayed on a computer screen for 1000 ms. On a “STOP” trial (25% of the 100 trials), the participant attempted to stop their response when a stop signal (a vertical arrow) appeared shortly following a “GO” stimulus. On these “STOP” trials, the time interval of 250 ms between “GO” signal and “STOP” signal onsets (e.g., stop signal delay) changed systematically according to each participant's performance. It became 50 ms longer after each successful stopping performance, making it harder to inhibit, and 50 ms shorter after each unsuccessful inhibition, making it easier to inhibit. The staircase algorithm ensured that the task was equally challenging and difficult for each individual, providing approximately 50% successful and 50% unsuccessful inhibition trials. The stop signal delay was calculated for each “STOP” trial. The stop signal reaction time (SSRT) was computed for individual subjects by subtracting the mean stop signal delay from the mean “GO” trial RT. Each “STOP” stimuli was displayed for 1000 ms—(current stop signal delay); thus the “GO” stimuli presentation time was equal to the time remaining from the aforementioned “STOP” difference from 1000 ms (Figure 1). A mean ITI was randomly jittered between 1.6, 1.7, 1.8, and 1.9 s to optimize statistical efficiency.

Participants were instructed to respond as fast as possible with a left or right key press (using index and middle fingers of the right hand) while maintaining a high level of accuracy. Responding quickly to the “GO” stimulus was emphasized by explaining to the participants that they were not to delay their response in anticipation of the stop signal, as it would not always be possible to withhold their response after detection of the stop signal. This was reinforced by showing participants their mean RT to the “GO” trials following each block of 100 trials, along with the message, “The fastest average RT for your age group is currently 422 ms, so try to reach or beat it!” This time of 422 ms was the fastest RT for a pilot group of 5 participants (data not presented). Participants practiced 80 trials of the stop signal task, then performed 6 blocks of 100 trials for the study. Participants also performed 100 trials of just the “GO” task (no “STOP” signals presented) to assess baseline RT behavior (RT baseline task). This task was performed separately from the other stop signal task blocks (always prior to any stop signal blocks), and also had a jittered ITI to match all methodological parameters used in the task excluding the presence of “STOP” trials.

### Behavioral analysis approach

The outcome of a single trial fell into one of three categories: go trials (GO) on which no stop signal appears, FI trials in which a stop signal appears but a response is still made, and SI trials in which a stop signal appears and no response is made. To evaluate the stop signal after-effect, all “GO” trials were divided into three different bins based on whether they were preceded by a GO trial (pGO), a FI trial (pFI), or a SI trial (pSI) (Figure 1). Trials for pGO, pSI, and pFI were also stratified by ITI duration to evaluate whether this jittered time interval affected subsequent RTs.

As previously described, Verbruggen et al. (2008) reported RT differences associated with the directional congruency of the subsequent “GO” trial arrow direction between pSI/pFI and pGO trials. The logic employed by these researchers was that if between-trial control adjustments are being made after successful inhibition trials, then one should observe longer pSI vs. pGO RTs regardless of whether the “GO” stimulus from trial *n* – 1 is repeated. Alternatively, if these after-effect following successful response inhibition are driven by repetition priming, then pSI RTs should be longer than pGO RTs *only* for trials where the direction of the “GO” stimulus repeats (see also Mayr et al., 2003) which would also argue against the Rieger and Gauggel (1999) persistence of inhibition interpretation. Thus, we also further stratified the pGO, pSI, and pFI trials by whether or not the directionality of the “GO” stimuli (i.e., pointing left or right) during the pGO/pSI/pFI trials were congruent with the previous “GO” stimuli. For example, if the GO stimulus in trial “*n* - 1” was a left pointing arrow and the GO stimulus in trial “*n*” was a right pointing arrow, then the stimulus in trial “*n*” was considered incongruent.

### EEG recording and data preprocessing

Participants were seated in an armchair in a dark room with the screen ~85 cm from the participants' eyes. Neural data were recorded with a BioSemi ActiveTwo 64-channel EEG acquisition system in conjunction with BioSemi ActiView software (Cortech-Solutions). Signals were amplified and digitized at 1,024 Hz with a 16-bit resolution. All electrode offsets were <25 kΩ. Anti-aliasing filters were used and data were band-pass filtered between 0.01 and 100 Hz during data acquisition. Preprocessing was conducted using Analyzer software (Brain Vision, LLC). Eye-movements artifacts were removed through an independent components analysis (ICA). The raw EEG-data were referenced to an average reference off-line and time-locked to stimulus onset for each trial type (“GO” stimulus for pGO, pSI, pFI). Trials were further cleaned of excessive peak-to-peak deflections, amplifier clipping, or other artifacts using a voltage threshold of 75 mV. Epochs (−3000 to +1000 ms, to encompass the previous trial and subsequent “GO” trial) for each trial type were time locked to the “GO” stimuli (see Figure 1).

### Channel/frequency selection

In attempt to narrow the focus of our subsequent analyses, we chose to focus on specific frequency bands at the C3, FCz, and F6 electrodes, as previous work has identified motor-related inhibitory activity at each of these electrodes (or their underlying regions) within certain frequencies. For example, the C3 electrode has been regularly used to examine inhibition-related processes originating near the motor cortex within the alpha frequency band (Serrien et al., 2005; Moore et al., 2006; Yamanaka and Yamamoto, 2010; Serrien and Sovijarvi-Spape, 2013). Theta-related activity near the FCz electrode has also been regularly examined given its proximity to premotor regions and associations with motor inhibition (Trujillo and Allen, 2007; Cavanagh et al., 2009; Brier et al., 2010; Yamanaka and Yamamoto, 2010; Liang et al., 2012; Müller and Anokhin, 2012). Finally, beta-related activity near the F6 electrode has been frequently interrogated with respect to right-lateralized stopping-related responses near this region with the stop-signal task (Serrien et al., 2005; Schmajuk et al., 2006; Liang et al., 2012; Swann et al., 2012) as well as with increased phase locking associated with “switch” trials (Gladwin et al., 2006; Serrien, 2009; Tallet et al., 2009). While the present analysis was driven by apriori hypotheses focusing exclusively on the frequencies associated with certain regions/electrodes in terms of motoric inhibition, we report the findings of the same analyses for all electrode/frequency combinations in an effort to provide full disclosure given that other studies have also associated certain frequencies at different regions with inhibition-related processes.

### IEC and ITC analyses

We examined IEC and ITC to test the phase consistency between (IEC) and within (ITC) electrodes for each condition (pGO, pSI, pFI) for each frequency band. These trials were convolved using EEGLAB's complex Morlet wavelet decomposition (Delorme and Makeig, 2004) to resolve frequencies from 4 to 65 Hz to calculate phase for each trial. Phase locking values (PLVs) for both IEC and ITC were computed by measuring the inter-trial variability of the phase difference at each time–frequency point (Lachaux et al., 1999). This procedure yields a PLV measure bound from 0 to 1 such that 0 represents random phase differences across trials while 1 indicates a consistent phase difference. For IEC, this involved calculating PLVs between our “seed” electrode/frequency (i.e., F6 in the beta band, C3 in the alpha band, FCz in the theta band) and all other electrodes. After calculating coherence from each of our three primary electrodes of interest to all other electrodes, we then created a global index of IEC for each frequency band by calculating the mean PLV to all electrodes for each condition (cf. Trujillo et al., 2005). For ITC, this involved calculating PLVs across trials at these seed electrodes. Within-subject differences in trial numbers were accounted for using a standardized bootstrap method (1000 permutations).

### Statistical analysis approach

We examined IEC and ITC for each condition (pGO, pSI, pFI) at each electrode within each frequency band at three distinct time periods. First, we examined the patterns of coherence prior to the “GO” trial stimulus onset during the prestimulus interval (−1000 to 0 in 100 ms intervals) using a condition × time window ANOVA at each electrode and frequency. Next, we examined the coherence patterns immediately following the moment of stimulus presentation (visual interrogation revealed peak activity to be centered between 0 and 200 ms). Finally, we examined coherence centered around the “GO” response using each individual's mean RT as the median and their own standard deviation as the window of interest. Follow-up contrasts were performed to further characterize any interactions observed, with a Greenhouse-Geisser correction utilized when assumptions of sphericity were not met. Planned contrasts for each frequency-associated electrode between each trial type were used to uncover any potential relationship(s) exhibiting a similar pattern to the behavioral findings. Furthermore, while our analyses were focused within these three different time periods, our motivation for this study was inherently driven by those results associated within the ITI. Thus, we report on observed activity following stimulus presentation but did not have any apriori hypotheses regarding patterns of activity at these time points.

## Results

### Behavioral results

**Figure 2:**
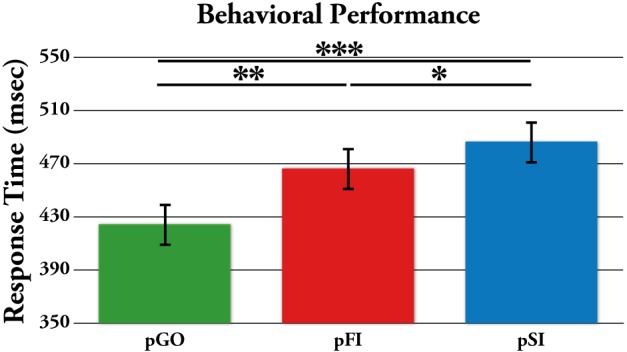
**Mean response time (RT) for each trial type, with the standard error of the mean represented as error bars.** pGO: GO trial following a GO trial. pSI: GO trial following a successful inhibition trial. pFI: GO trial following a failed inhibition trial. ^*^p < 0.05, ^**^p < 0.001.

Performance data describing the stop signal task are presented in Table 1. The effect of ITI and condition on RTs was tested using a Two-Way ANOVA of ITI (1.6 s, 1.7 s, 1.8 s, 1.9 s) × condition (pGO, pSI, pFI), revealing main effects of ITI [*F*_(3, 51)_ = 6.3, *p* < 0.01] and condition [*F*_(2, 34)_ = 19.3, *p* < 0.001], but no condition X ITI interaction [*F*_(6, 102)_ = 1.46, *p* = 0.20; see Supplementary Figure 1]. A within-subjects contrast of ITI for linear effects was significant [*F*_(1, 17)_ = 16.31, *p* = 0.001], indicating that RTs decreased as the ITI decreased in length from 1.9 to 1.6 s across all conditions. Follow-up *t*-tests examining the main effect of condition revealed that the RTs for the pGO condition (418 ms ± 19) were significantly faster than both the pFI (459 ms ± 16, *t* = 4.22, *p* < 0.01) and the pSI (477 ms ± 19, *t* = 4.96, p < 0.001) conditions, with the pSI trials being slower than the pFI trials (*t* = 2.2, *p* < 0.05; see Figure 2). Thus, there was a significant influence on RT based upon the identity of the previous trial type, with longer ITIs corresponding with longer RT in general.

**Table 1:** **Stop signal behavioral measures (Mean and Standard Error)**.

	**Mean RT**	**Stimulus-repeating**	**Non-repeating**
pGO	424 (18)	440 (15)	429 (15)
pSI	486 (17)	505 (16)	470 (17)
pFI	466 (15)	473 (13)	447 (15)
	**Stop signal delay**	**SSRT**	
pSI	181 (13)	304 (10)	
pFI	226 (14)	240 (6)	

To test whether these effects were driven by between-trial control adjustments vs. repetition-priming effects (Verbruggen et al., 2008), a separate condition (pGO, pSI, pFI) × directional congruency of the “GO” arrows on trial *n* − 1 and *n* (same vs. different direction) ANOVA revealed incongruent directionality of the “GO” stimuli vs. the preceding trial led to faster RT in a differential fashion for each condition [*F*_(2, 34)_ = 4.14, *p* = 0.024]. Follow-up analyses revealed a significant difference between pSI and pGO trials regardless of whether they were directionally congruent [*t* = 6.00, *p* < 0.001] or incongruent (*t* = 4.44, *p* < 0.001), with the same pattern observed for pFI and pGO trials (for each comparison *t* > 2.60, *p* < 0.018) as well as pSI and pFI trials (for each comparison *t* > 3.03, *p* < 0.007). Unlike Verbruggen et al. (2008), whose repetition-priming interpretation was based upon no difference being present between pSI and pGO trials on incongruent trials, the directional differences observed here suggests the involvement of between-trial control adjustments.

### Neural analyses

The following neural analyses focused on IEC and ITC activity within specific frequency bands at the C3 (alpha), FCz (theta), and F6 (beta) electrodes in accord with previous work describing this type of activity at these electrodes (or their underlying regions) within certain frequencies bands. In all subsequent analyses (except those stating otherwise), we observed the same pattern of significance when comparing pGO and pSI as when comparing pGO and pFI (see Supplementary Tables 1, 2 for an overview of all subsequent analyses and findings, and Supplementary Figures 2–7 for all other ITC frequency/electrode combinations not driven by apriori hypotheses). Thus, in describing these results, we combined the description of these analyses (even though their analyses were performed separately) as indicated by the pSI/pFI term. For all of the analyses examining the prestimulus period, the factor of time window (100 ms intervals from −1000 to 0) was included in each respective ANOVA; however, there were no interactions involving this factor in any analyses.

#### IEC during the inter-trial interval

**Figure 3:**
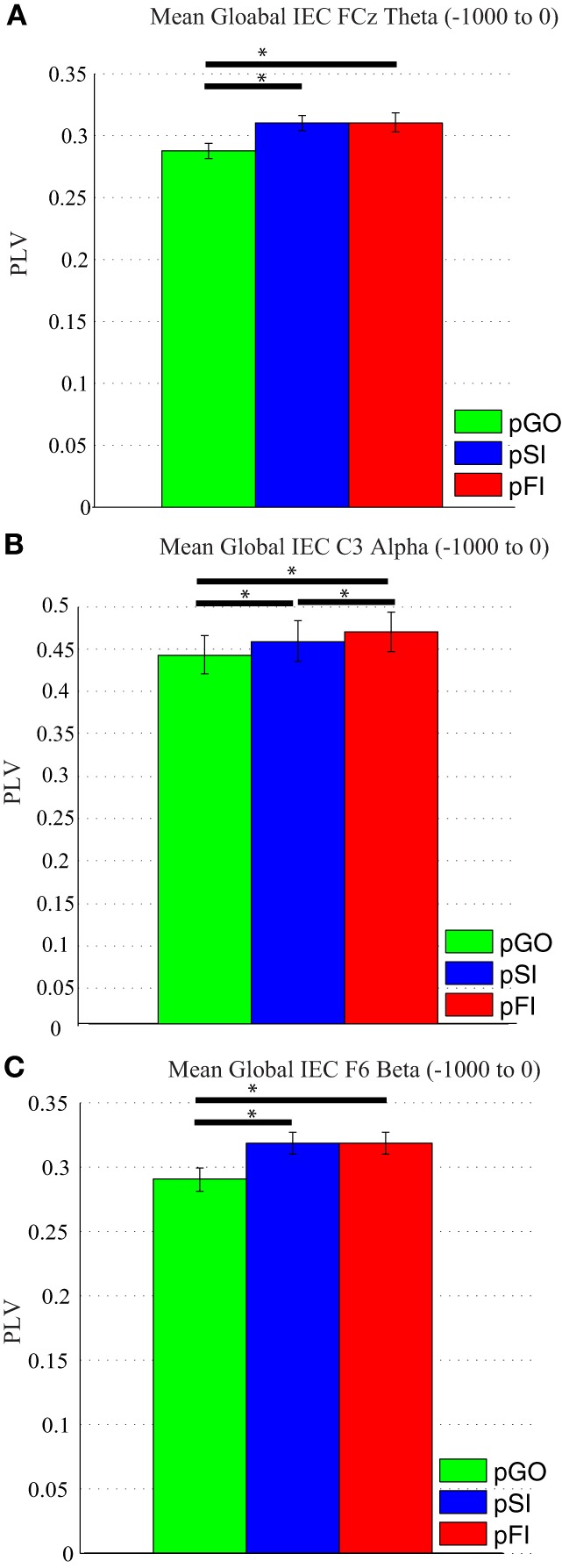
**Mean global inter-electrode coherence (IEC) over the −1000 to 0 ms interval. (A)** Mean theta IEC from the FCz electrode to all other electrodes. **(B)** Mean alpha IEC from the C3 electrode to all other electrodes. **(C)** Mean beta IEC from the F6 electrode to all other electrodes. ^*^p < 0.05.

A Two-Way ANOVA involving time window (10) and condition (3) for theta activity at the FCz electrode revealed a main effect of condition [*F*_(2, 34)_ = 15.39, *p* < 0.0001]. Comparing the pSI/pFI and pGO conditions, there was an effect of condition with pSI/pFI showing greater IEC than pGO [*F*_(1, 17)_ > 21.20, *p* < 0.0001 for each comparison], but no effect of condition between pSI and pFI trial types [*F*_(1, 17)_ = 2.43, *p* = 0.13; see Figure 3; for result of other frequency bands at this electrode, see Supplementary Table 1].

Using the same approach for alpha activity at the C3 electrode, a main effect of condition was present [*F*_(2, 34)_ = 16.27, *p* < 0.001]. Comparing the pSI/pFI and pGO conditions, there was an effect of condition, with pSI/pFI showing greater IEC than pGO [*F*_(1, 17)_ > 10.80, *p* < 0.007 for each comparison], with an effect of condition between pSI and pFI trial types (pFI > pSI; *F*_(1, 17)_ = 6.63, *p* = 0.02; see Figure 3; for result of other frequency bands at this electrode, see Supplementary Table 1).

Using the same approach for beta activity at the F6 electrode, a main effect of condition was present [*F*_(2, 34)_ = 11.73, *p* < 0.001]. Comparing the pSI/pFI and pGO conditions, there was an effect of condition, with pSI/pFI showing greater IEC than pGO [*F*_(1, 17)_ > 11.80, *p* < 0.005 for each comparison], but no effect of condition between pSI and pFI trial types [*F*_(1, 17)_ = 2.64, *p* = 0.12; see Figure 3; for result of other frequency bands at this electrode, see Supplementary Table 1]. Given that the directional differences observed within the behavioral data suggest the involvement of between-trial control adjustments, these IEC findings would support this interpretation as both “STOP” trial types demonstrated a difference from pGO trials in terms of greater global coherence. The exact same pattern of effects were also observed when restricted to only the electrodes of interest (e.g., FCz, C3, F6), confirming a conditional change in global coherence during the “STOP” vs. “GO” trial types.

#### IEC after “GO” stimulus onset and centered around the “GO” response

For each time period, a similar pattern emerged: there was greater pSI/pFI than pGO IEC [*F*_(1, 17)_ ≥ 7.97, *p* ≤ 0.012 for each comparison and time period], but no difference present between pSI and pFI trial types [*F*_(1, 17)_ ≥ 2.43, *p* ≤ 0.14 for each comparison and time window; see Supplementary Table 1]. Thus, as with the ITI IEC findings, both “STOP” trial types demonstrated a difference from pGO trials that was congruent with the behavioral observed with these same trial types. As with the ITI findings, the exact same pattern of effects were also observed when restricted to only the electrodes of interest as during the ITI analysis.

#### ITC during the inter-trial interval

**Figure 4:**
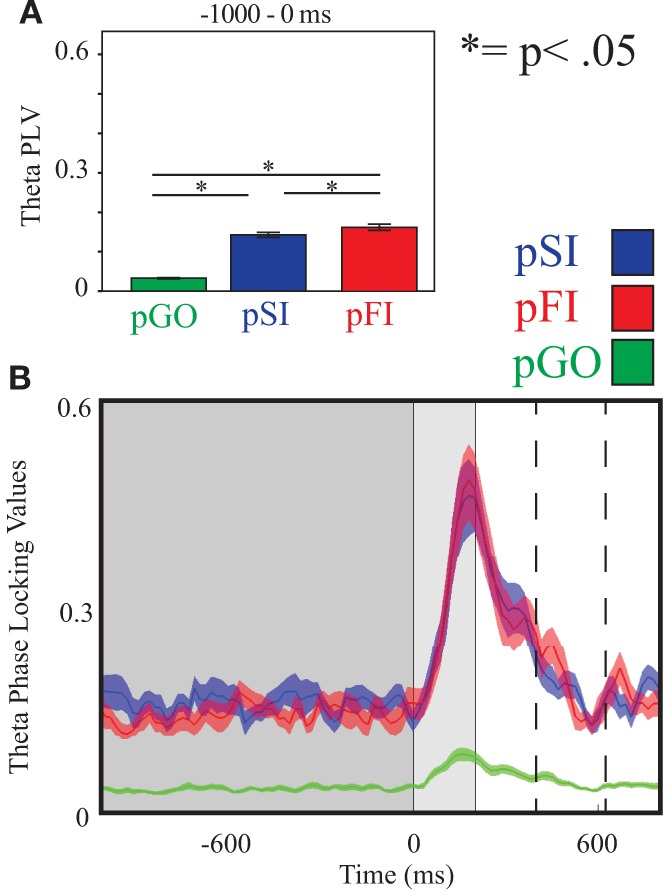
**Theta inter-trial coherence (ITC) at electrode FCz. (A)** Bar graph displaying mean ITC averaged over the −1000 to 0 ms interval, with 0 ms as GO stimulus onset. **(B)** Line plot illustrating ITC from −1000 to +1000 ms, with the dark gray highlighting the ITI, the light gray bar after 0ms highlights maximal coherence following stimulus onset, and the dashed lines indicating ITC centered around the “Go” response.

Using the same Two-Way ANOVA analysis approach described above for IEC, theta activity at the FCz electrode again revealed a main effect of condition [*F*_(2, 34)_ = 246.00, *p* < 0.001]. Comparing the pSI/pFI and pGO conditions, there was an effect of condition, with pSI/pFI showing greater ITC than pGO [*F*_(1, 17)_ > 393.00, *p* < 0.001 for each comparison]. Comparing pSI and pFI, there was an effect of condition [pFI > pSI; *F*_(1, 17)_= 5.15, *p* = 0.03; see Figure 4; for result of other frequency bands at this electrode, see Supplementary Table 2].

**Figure 5:**
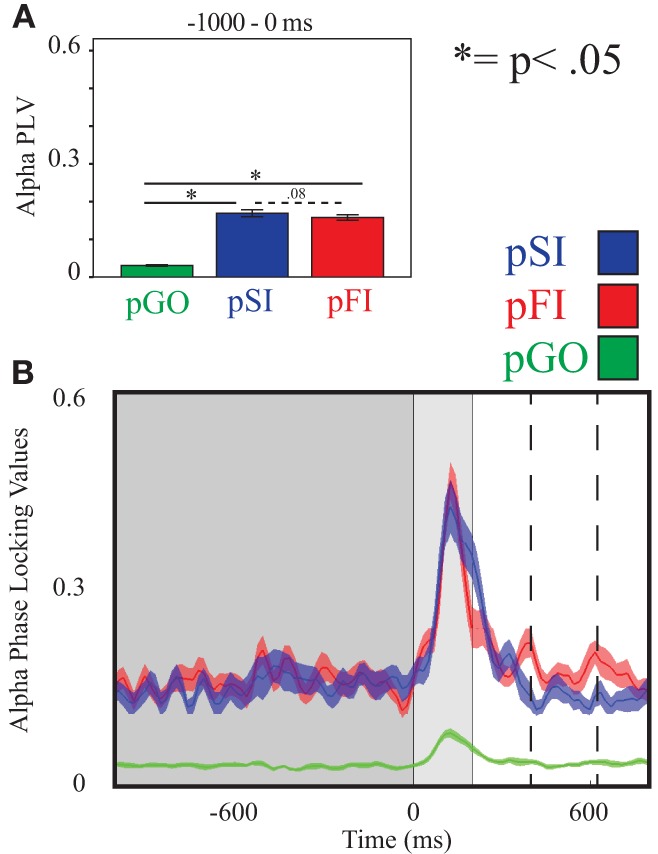
**Alpha inter-trial coherence (ITC) at electrode C3. (A)** Bar graph displaying mean ITC averaged over the −1000 to 0 ms interval, with 0ms as GO stimulus onset. **(B)** Line plot illustrating ITC from −1000 to +1000 ms, with the dark gray highlighting the ITI, the light gray bar after 0 ms highlights maximal coherence following stimulus onset, and the dashed lines indicating ITC centered around the “Go” response.

Using the same approach for alpha activity at the C3 electrode, there was an effect of condition [*F*_(2, 34)_ = 250.00, *p* < 0.001], with follow up analyses comparing pSI/pFI and pGO also revealing an effect of condition [*F*_(1, 17)_ > 419.00, *p* < 0.001 for each comparison]. Between pSI and pFI trial types, there was a trend again toward significance [pSI > pFI; *F*_(1, 17)_ = 3.36, *p* = 0.08; see Figure 5; for result of other frequency bands at this electrode, see Supplementary Table 2].

**Figure 6:**
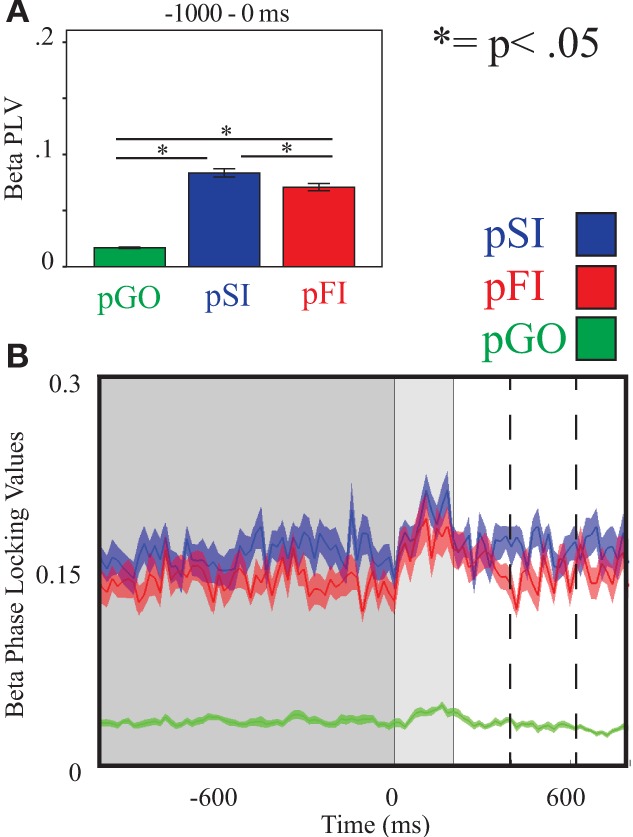
**Beta inter-trial coherence (ITC) at electrode F6. (A)** Bar graph displaying mean ITC averaged over the −1000 to 0 ms interval, with 0ms as GO stimulus onset. **(B)** Line plot illustrating ITC from −1000 to +1000 ms, with the dark gray highlighting the ITI, the light gray bar after 0 ms highlights maximal coherence following stimulus onset, and the dashed lines indicating ITC centered around the “Go” response.

Using the same approach for beta activity at the F6 electrode, there was an effect of condition [*F*_(2, 34)_ = 234.00, *p* < 0.001] with follow up analyses comparing pSI/pFI and pGO yielding an effect of condition in each case [*F*_(1, 17)_ > 366.00, *p* < 0.001 for each comparison]. Comparing pSI and pFI trial types revealed an effect of condition, with greater pSI activity [*F*_(1, 17)_ = 9.80, *p* < 0.01; see Figure 6; for result of other frequency bands at this electrode, see Supplementary Table 2] Thus, we confirmed our hypotheses regarding the influence of regionally-specific alpha and beta ITC during the ITI as function of different trial types that mirrored the observed pSI > pFI > pGO behavioral effect.

#### ITC after “GO” stimulus onset

As above, analyses were performed comparing conditions within a particular frequency band at each electrode. For each comparison, the same pattern was observed: there was greater pSI than pGO ITC [*F*_(1, 17)_ > 150.00, *p* < 0.001], greater pFI than pGO coherence [*F*_(1, 17)_ > 94.00, *p* < 0.001], but no difference between pSI and pFI [*F*_(1, 17)_ ≤ 2.72, *p* > 0.12; see Supplementary Table 2]. Thus, as with the ITI IEC findings, both “STOP” trial types demonstrated a difference from pGO trials that was congruent with the behavioral observed with these same trial types.

#### ITC centered around the “GO” response

Examining theta ITC at electrode FCz centered around the moment of response to the subsequent “GO” stimuli, we observed an effect of condition [*F*_(2, 34)_ = 20.60, *p* < 0.001]. Follow up analyses indicated that ITC was greater for pSI/pFI than pGO trials [*F*_(1, 17)_ > 33.10, *p* < 0.001 for each comparison]. However, there were no differences when comparing pSI and pFI [*F*_(1, 17)_ = 1.42, *p* > 0.20; for result of other frequency bands at this electrode, see Supplementary Table 2].

Examining alpha ITC at C3, an effect of condition was again observed [*F*_(2, 34)_ = 34.21, *p* < 0.001]. Comparing pSI/pFI and pGO indicated greater pSI/pFI coherence [*F*_(1, 17)_ > 16.72, *p* < 0.001 for each comparison], with greater alpha ITC for pSI vs. pFI trial types [*F*_(1, 17)_ > 6.97, *p* < 0.05; for result of other frequency bands at this electrode, see Supplementary Table 2]

Finally, examining beta ITC at electrode F6 revealed an effect of condition [*F*_(2, 34)_ = 17.30, *p* < 0.001]. Greater pSI/pFI than pGO ITC was evidenced [*F*_(1, 17)_ > 15.70, *p* < 0.001 for each comparison], with greater beta ITC during pSI vs. pFI trials [*F*_(1, 17)_ = 4.82, *p* < 0.05; for result of other frequency bands at this electrode, see Supplementary Table 2]. Thus, examination of inhibition-related ITC centered around the moment of response showed the same pattern of effects as seen during the ITI for the F6 electrode, but no clear similarities for the other electrodes or periods tested, nor (most importantly) with the observed behavioral effects.

## Discussion

Both pSI and pFI trials were slower than pGO trials, replicating previous stop signal after-effect studies (Rieger and Gauggel, 1999; Verbruggen et al., 2005a; Li et al., 2008; Verbruggen and Logan, 2008). However, we also observed (i) pSI trials being slower than pFI trials, (ii) a general effect of ITI on RTs, and (iii) behavioral evidence supporting a between-trial control adjustment interpretation over a repetition-priming explanation. Our neural analyses revealed increased IEC and ITC for “STOP” vs. “GO” trial types, indicative of a difference in cognitive processing for these inhibitory-laden trial types. Critically, the observed pSI > pFI > pGO pattern of behavior was matched only by the ITC analysis within the beta and alpha frequency bands during the ITI at the apriori specified electrodes. Here we describe how these behavioral and neural findings are indicative of between-trial control adjustments involved with both task-set switching and motor inhibition processes during these stop signal after-effects.

### Behavioral interpretations

The longer RTs following “STOP” (pSI, pFI) vs. “GO” (pGO) stimuli support the idea of motor inhibition processes persisting from trial “*n* − 1” to trial “n,” as this ordering (i.e., pSI > pFI > pGO) would fit the theoretically perceived amount of inhibition-related processes engaged in each condition. Verbruggen et al. (2008) argument for these types of findings reflecting repetition priming effects rather than between-trial control adjustments was based upon the idea that if successful response inhibition is due primarily to repetition priming, then pSI should be longer than pGO *only* for stimulus-repetition trials vs. non-repeating stimulus trials (see also Upton et al., 2010). However, unlike Verbruggen et al. (2008), we did evidence a significant effect for both stimulus-repeating and non-repeating trials, indicative of between-trial control adjustments. Given that similar findings have demonstrated slowing for both correct and incorrect trials following the presentation of infrequent stimuli (Notebaert et al., 2009), the type of adjustment found here agrees with the idea of a shift in strategy following the “STOP” stimuli in line with between-trial control adjustments.

The infrequent nature of stop signals here (appearing on 25% of trials) implies that the likelihood of two stop signals occurring sequentially to be only 6.25%, a percentage that participants could have inferred (but was not directly probed for here) but seems unlikely. As such, the presence of a stop signal in trial “*n* − 1” would theoretically elicit a strategic shift toward making a “GO” response in trial “*n*” moreso than a shift toward making another “STOP” response^1^. Thus, this congruency analysis suggests a conditional strategic effect may be in play when presented with different trial types. It should be noted that unlike the previously mentioned stop signal after-effect studies, longer RTs for pSI vs. pFI trials were also observed. This discrepancy may stem from the ITI jittering approach used here, as the other studies each used a fixed ITI length (Rieger and Gauggel, 1999). Given that changes in ITI have been shown to affect RTs when switching between conditional trial types (Altmann, 2004a,b; Monsell and Mizon, 2006), the variable ITI appears to have influenced not only the difference between “STOP” and “GO” trials, but also revealed the subtle difference between pSI and pFI trial types.

The behavioral analyses are in agreement with the idea that participants may have been anticipating a switch from a “STOP” trial (on trial *n* − 1) to a “GO” trial (trial *n*), leading to these after-effects. Task switching, which involves the active reconfiguration of mental resources when task requirements change (Logan and Delheimer, 2001; Logan and Gordon, 2001; Monsell, 2003; Yeung et al., 2006; Vandierendonck et al., 2010) is known to produce slower RTs in the form of switch costs (Monsell, 2003). This interpretation, which is also in line with the “task-set inertia” hypothesis (Allport et al., 1994), is consistent with the theory that these stop-signal after-effects reflect participants strategically anticipating and subsequently reconfiguring their task goals following both pSI and pFI trials (unlike pGO trials, where a “GO” stimuli was repeated). Evidence for this interpretation is borne out in the neural data, described below.

### Neural findings reflecting motor inhibition processes

The two neural measures used here, IEC and ITC, each showed similar patterns to the behavioral findings: a conditional increase in phase synchrony for both pSI and pFI trial types vs. pGO trials, such that greater coherence (that is, *less* variability (or *more* consistent) engagement) associated with motor inhibition processes was observed following a “STOP” trial. These findings suggest that the focused engagement of motor inhibition processes persists during the ITI, and having to reset the synchronization of neural oscillations within specific cortical areas from an “inhibitory” state to a “action” state (that is, changing from “STOP” to “GO”)^2^ underlies the observed behavioral slowing in a manner that is congruent with the “task-set inertia” hypothesis. This interpretation agrees with work describing that the networks involved in mediating stop signal inhibition were also identified during task switching (Kenner et al., 2010), and other studies that reported increased coherence when switching between task sets in the beta (Gladwin et al., 2006; Serrien, 2009; Tallet et al., 2009) and alpha (Serrien et al., 2004; Serrien and Sovijarvi-Spape, 2013) frequency bands. Similarly, fMRI studies have described the engagement of lateral prefrontal regions when overcoming residual cognitive inhibition (Dreher and Berman, 2002; Dreher et al., 2002), with this activity being related to the re-engagement of a previous task set within the same paradigms (Dreher and Berman, 2002). Indeed, recent IEC findings by Müller and Anokhin (2012) have also suggested increased task demands during response inhibition require stronger phase synchronization, with this phase locking indicative of an anticipatory switching process (Gladwin et al., 2006). Thus, the observed pattern of global IEC suggests that regions associated with motor inhibition processes are communicating with a number of other areas as a network when switching from a “STOP” to a “GO” state, with greater synchronization between these regions contributing to the observed behavioral slowing following “STOP” trials.

However, while the IEC metric did not follow the observed pattern of behavior (pSI < pFI < pGO) that also distinguished between the “STOP” trial types, this effect was present for the ITC analyses. We hypothesized that ITC activity would be best observed during the ITI within certain frequency bands nearest to stop-signal inhibition specific regions, with this activity reflecting greater local (as opposed to global) synchronization associated with motor inhibition processing. Under this premise, ITC differences between pSI and pFI trial types were found within the beta frequency band near the rIFG. Using the task-set inertia hypothesis as a framework, a pSI trial could be considered a “complete” switch as the “STOP” task was successfully performed on the previous trial, whereas a pFI trial would then be an “incomplete” switch trial. This interpretation agrees not only with the premise that increased cognitive demands, like task switching, call for greater coherence but also agrees with other task switching work that has also evidenced increased beta-band phase locking preceding switch trials (Gladwin et al., 2006; Serrien, 2009; Tallet et al., 2009). Given that rIFG activity has also shown modulation with stop signal success on the previous trial in fMRI studies (Li et al., 2008), these findings are suggestive of the prior engagement motor inhibition processes influencing switching between task sets which contributes to a RT slowing.

These interpretations are supported by the related alpha ITC (and IEC) findings near the motor cortex during the ITI. The synchronization of alpha power at motor regions has been associated with the inhibitory control (Hummel et al., 2002; Klimesch et al., 2007) and task switching (De Jong et al., 2006). Most related to the present study, the pattern(s) of coherence observed here agree with previous studies utilizing alpha coherence measures to support the theory that task switching engages inhibitory processes to swap between task sets (Serrien et al., 2004; Serrien, 2009; Serrien and Sovijarvi-Spape, 2013). Swann et al. (2009) have previously identified the motor cortex as a downstream target of prefrontal regions with respect to both alpha (and beta) when engaging motor inhibition processes. In agreement with this interpretation, we observed a trend toward greater response-centered alpha ITC phase-locking for pSI vs. pFI trials, as well as faster RT during pFI vs. pSI trials, suggesting that pSI trials had inhibitory control processes engaged to a greater extent than pFI trials.

Consequently, we also observed greater theta ITC for the pFI vs. pSI trials at the FCz electrode during the ITI. Greater theta ITC nearest midline frontal regions has previously been associated with voluntary response inhibition processes (Brier et al., 2010; Yamanaka and Yamamoto, 2010; Müller and Anokhin, 2012), with theta- (and beta-) driven coherence amongst the rIFG, preSMA, and primary motor cortex suggested to be critical for inhibitory control during the stop signal task (Liang et al., 2012). However, it should also be noted that theta-band power and oscillatory activity have been associated with conflict monitoring (Hanslmayr et al., 2008; Cavanagh et al., 2009; Nigbur et al., 2012), suggesting that the observed conditional differences may also reflect a combination of multiple cognitive processes. This interpretation would agree with the present findings given that pFI trials would have greater conflict than pSI trials given the presence of an error on the preceding trial.

It is interesting, yet unclear, why these differential patterns of ITC between pSI and pFI trials were no longer present immediately following “GO” stimulus presentation, and inconsistent when examined around the moment of response. The influence of the recently encountered “STOP” trial type is seen to persist beyond the ITI, with the consistent finding of pSI = pFI > pGO for both the IEC and ITC within each frequency band immediately after stimulus presentation indicating of a common feature between these trial types (e.g., task set switching). However, it is likely that other cognitive factors like error monitoring (Carp and Compton, 2009; Nigbur et al., 2012) may be in play nearest the moment of response, potentially accounting for the inconsistencies between the neural effect and observed differences in behavior for each condition.

### Reconciliation of motor inhibition and task switching contributions

We propose the present findings are the product of two principal sources of slowing in the stop signal task: motor inhibition processes and a strategic decision implemented when switching between task sets. It is tempting to speculate that the conditional global IEC effects may better reflect the involvement of task switching processes, while the ITC results highlight the underlying motor inhibition processes engaged during each condition given the similarities to the observed behavior. However, confirming this interpretation would require further investigation as the present experimental design could not determine whether these outcomes are truly mutually exclusive. Nevertheless, the idea of conditional changes in phase synchrony near motor, pre-motor, and prefrontal regions agrees with elegant TMS work characterizing a functional interaction between pre-SMA, primary motor cortex, and the right IFG specific to action reprogramming trials (Neubert et al., 2010). Indeed, the pre-SMA has been shown to facilitate the correct action on switch trials (Mars et al., 2009) and has a critical relationship with the right IFG during action inhibition (Duann et al., 2009; Obeso et al., 2013). Thus, we cautiously speculate that the increased IEC/ITC observed during the ITI between pSI/pFI and pGO trial types is facilitating set switching by modulating the phase synchrony of activity between right IFG, pre-SMA, and primary motor cortex.

These strategic decisions appear to be influenced by the jittered ITI, which suggests why studies using a fixed ITI may have observed a different pattern of behavioral results. Unlike Verbruggen et al. (2008), whose Experiment 1a findings suggested that the observed slowing could be either task switching or repetition priming (which led to subsequent experiments validating their repetition priming interpretation), our behavioral findings were consistent with the task set switching perspective and subsequently guided our neural analyses. However, an open question that remains involves the differences in the neural correlates associated with repetition-priming and between-trial control adjustments, as the neural findings themselves cannot directly discount the possibility of repetition-priming without assessing directional congruency. The present experimental design resulted in a relatively small number of each directional trial type (the mean number of pSI & pFI congruent & incongruent trials was ~25 ± 5 each), providing only a modest signal-to-noise ratio for subsequent neural analyses of these repetition-priming effects. However, given that the behavioral results did not statistically support interrogating these directional effects, this analysis was not warranted here. Nevertheless, this proposed task-set switching interpretation is supported by the region-specific ITC that follows the observed behavioral finding (pSI > pFI > pGO).

Although these findings are limited in terms of their spatial resolution, they were driven by planned analyses focusing on regionally-specific activity patterns within certain frequency bands based on previous motoric inhibition work. Outside of these planned analyses, some of the other electrode/frequency combinations also showed inter-trial coherence that differentiated between the pSI and pFI trial types. However, these adjunct findings and their subsequent interpretations are not clearly supported in the motor inhibition literature; given that these findings were not hypothesis driven and the number of analyses performed, it is unlikely that their significance would survive any type of multiple comparisons correction. Rather, their inclusion is in the interest of full disclosure for researchers interested in the results at these regions using this type of analyses given the number of EEG stop-signal studies in recent years.

While the present study focused on coherence centered near prefrontal and motor cortex electrodes within beta, alpha, and theta frequency bands, these effects likely also involve a network of regions beyond the range of surface EEG spatial resolution used here. For example, areas within the basal ganglia have also been associated with the selection and inhibition of competing motor programs (Mink, 1996; Wichmann and Delong, 1996; Kropotov and Etlinger, 1999; Mink, 2006), including during task switching (Kenner et al., 2010). Future work that integrates these other regions and frequencies, especially in populations known to have deficiencies in task switching (ex. older adults: Kray and Lindenberger, 2000; Mayr and Kliegl, 2000; Kray et al., 2002; Adrover-Roig and Barcelo, 2010; Jimura and Braver, 2010) is warranted to extend the present findings, providing a more thorough characterization of these stop signal after-effects.

### Conflict of interest statement

The authors declare that the research was conducted in the absence of any commercial or financial relationships that could be construed as a potential conflict of interest.

## Footnotes

^1^While this interpretation would seemingly predict that the pSI RTs should be faster than the pGO RTs, the congruency delay described above, as well as the RT cost associated with making such a switch, precludes this from being the case.

^2^The reconfiguration view of task switching (Vandierendonck et al., 2010) is similar in premise to the task-set inertia hypothesis; however, given the design of the present study, we could not directly test this theory given that stimulus-response mapping did not change at any time during the task.
