# Supplementary material for: Reconciling the influence of task-set switching and motor inhibition processes on stop signal after-effects
Source: Front Psychol. 2013 Sep 24;4:649. doi: 10.3389/fpsyg.2013.00649 (PMC3781352; doi:10.3389/fpsyg.2013.00649)
Supplement: Supplementary file 1 [file Presentation1.PDF]

Supplementary Table 1. Summary of Inter-Electrode Coherence Analyses

Electrode	Frequency	Inter-Trial Interval	Stimulus Locked	Response Centered
**FCZ**	Theta	**pSI=FI>pGO**	pSI=FI>pGO	pSI=pFI>pGO
	Alpha	pSI=pFI>pGO	pSI=FI>pGO	pSI=pFI>pGO
	Beta	pSI>pFI>pGO	pSI=FI>pGO	pSI=pFI>pGO
**C3**	Theta	pSI>pFI>pGO	pSI=FI>pGO	pSI=pFI>pGO
	Alpha	**pFI>pSI>pGO**	pFI=SI>pGO	pSI=pFI>pGO
	Beta	pSI=FI>pGO	pSI=FI>pGO	pSI=pFI>pGO
**F6**	Theta	pSI=pFI>pGO	pSI=FI>pGO	pSI=pFI>pGO
	Alpha	pSI=pFI>pGO	pSI=FI>pGO	pSI=pFI>pGO
	Beta	**pSI=pFI>pGO**	pSI=FI>pGO	pSI=FI>pGO

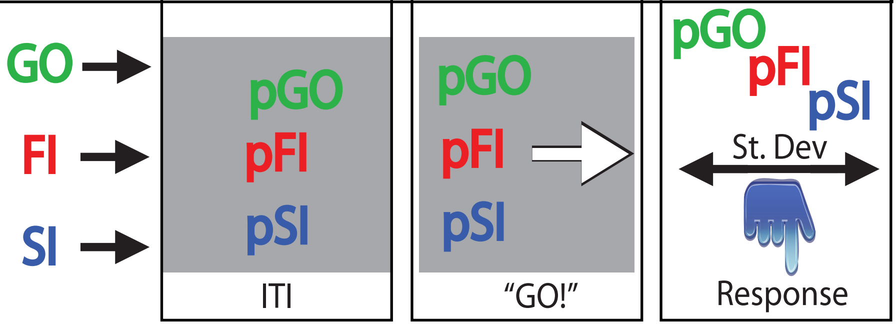

Supplemental Table 2. Summary of Inter-Trial Coherence Analyses

Electrode	Frequency	Inter-Trial Interval	Stimulus Locked	Response Centered
**FCZ**	Theta	**pFI>pSI>pGO**	pSI=FI>pGO	pSI=pFI>pGO
	Alpha	pSI=pFI>pGO	pSI=FI>pGO	pSI=pFI>pGO
	Beta	pSI>pFI>pGO	pSI=FI>pGO	pSI=pFI>pGO
**C3**	Theta	pSI>pFI>pGO	pSI=FI>pGO	pFI>pSI>pGO
	Alpha	**pSI≥pFI>pGO**	pSI=FI>pGO	pFI>pSI>pGO
	Beta	pSI>pFI>pGO	pSI=FI>pGO	pSI=pFI>pGO
**F6**	Theta	pSI=pFI>pGO	pSI=FI>pGO	pSI=pFI>pGO
	Alpha	pSI>pFI>pGO	pSI=FI>pGO	pSI>pFI>pGO
	Beta	**pSI>pFI>pGO**	pSI=FI>pGO	pSI>pFI>pGO

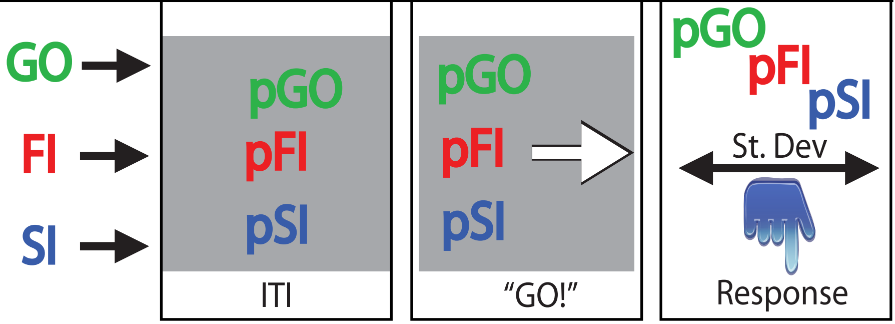

- Bolded comparisons indicate those planned channel/frequency comparisons.
- > represents p< .05; ≥ represent p= .08

Supplemental Figure 1

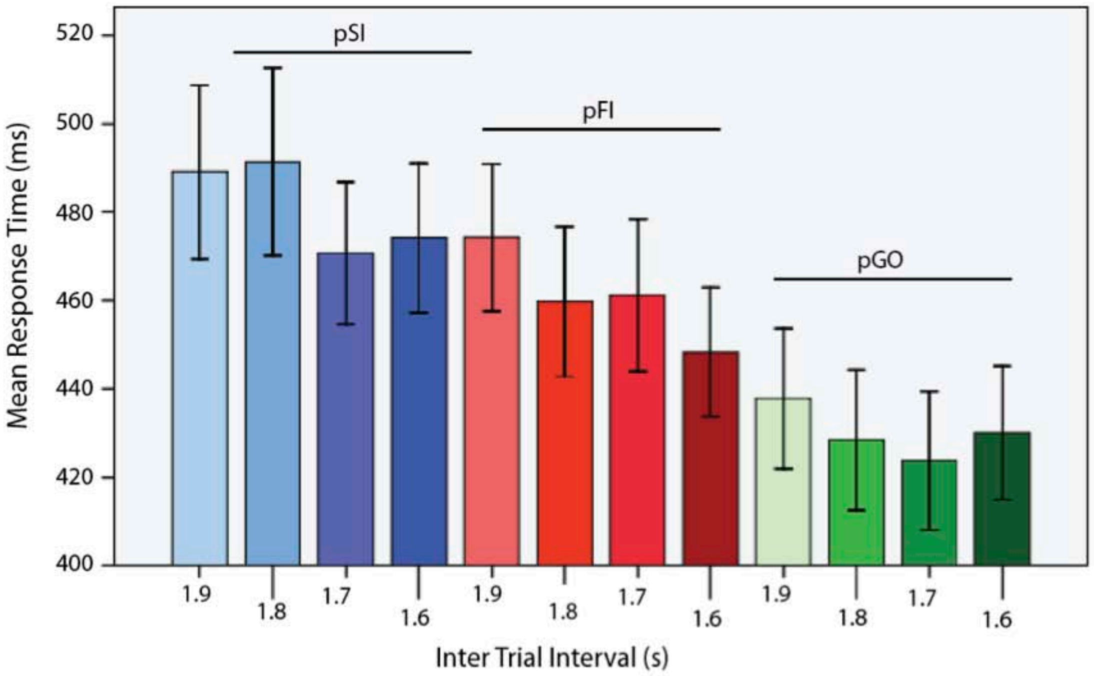

Supplemental Figure 1. Mean RTs (± SE) stratified by condition and inter-trial interval (ITI) length.

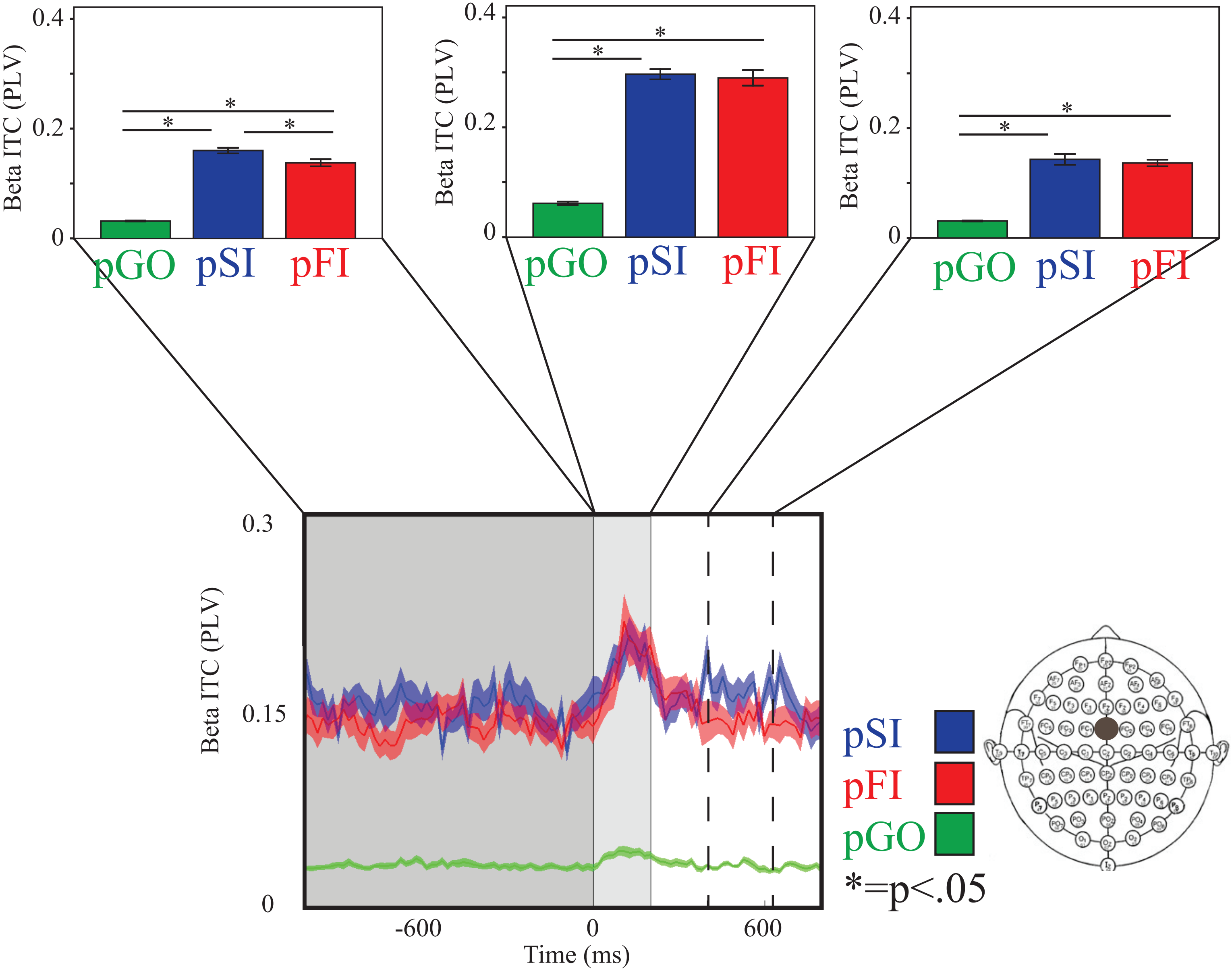

Supplementary Figure 2. Beta ITC at electrode FCZ. A) Bar graph displaying mean beta ITC averaged over the -1000 to 0ms interval, with 0ms as GO stimulus onset. B) Bar graph displaying the maximum ITC following GO stimulus onset (from 0 to 200ms). C) Beta ITC relative to the mean response time for each trial type.

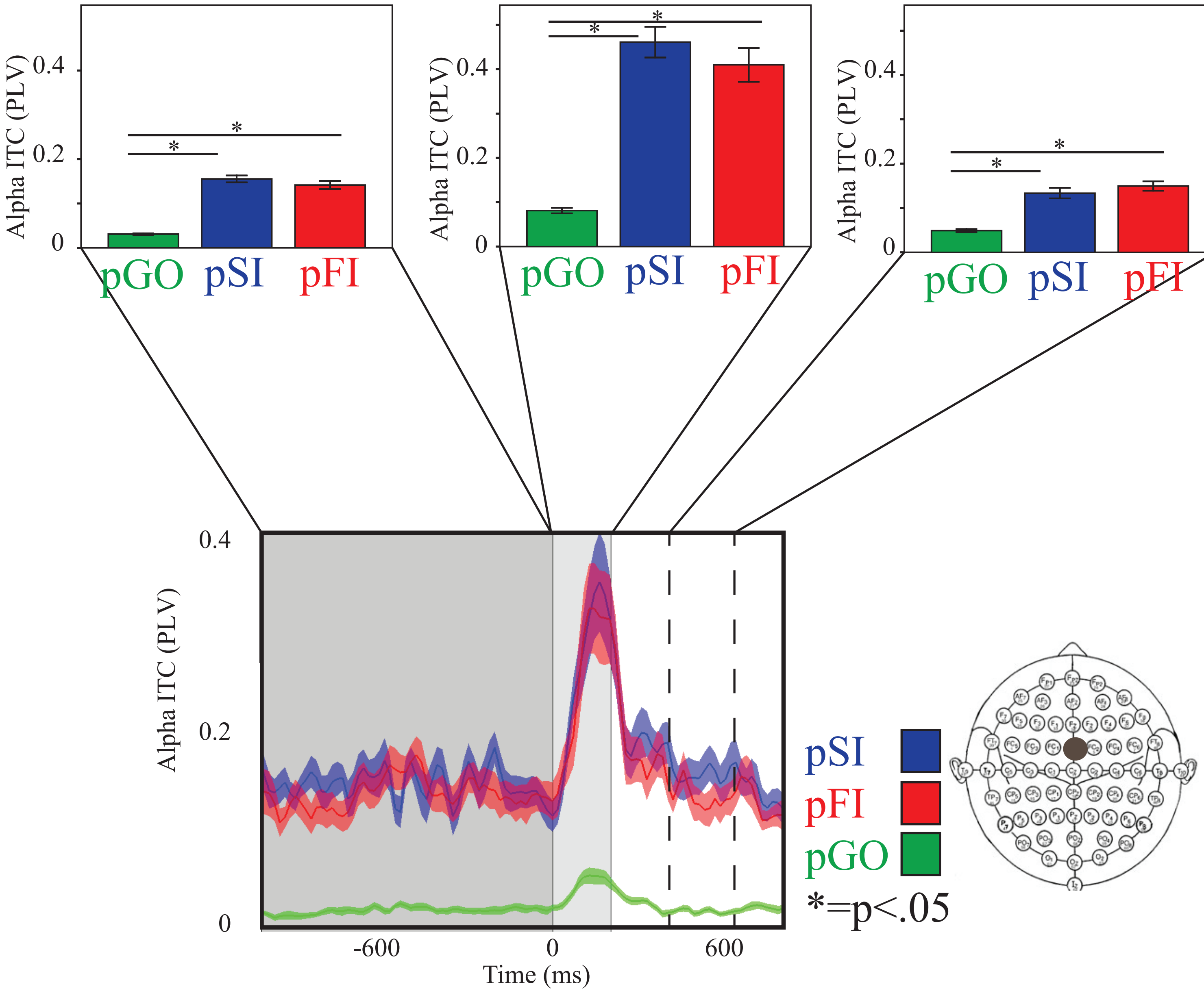

Supplementary Figure 3. Alpha ITC at electrode FCZ. A) Bar graph displaying mean alpha ITC averaged over the -1000 to 0ms interval, with 0ms as GO stimulus onset. B) Bar graph displaying the maximum ITC following GO stimulus onset (from 0 to 200ms). C) Alpha ITC relative to the mean response time for each trial type.

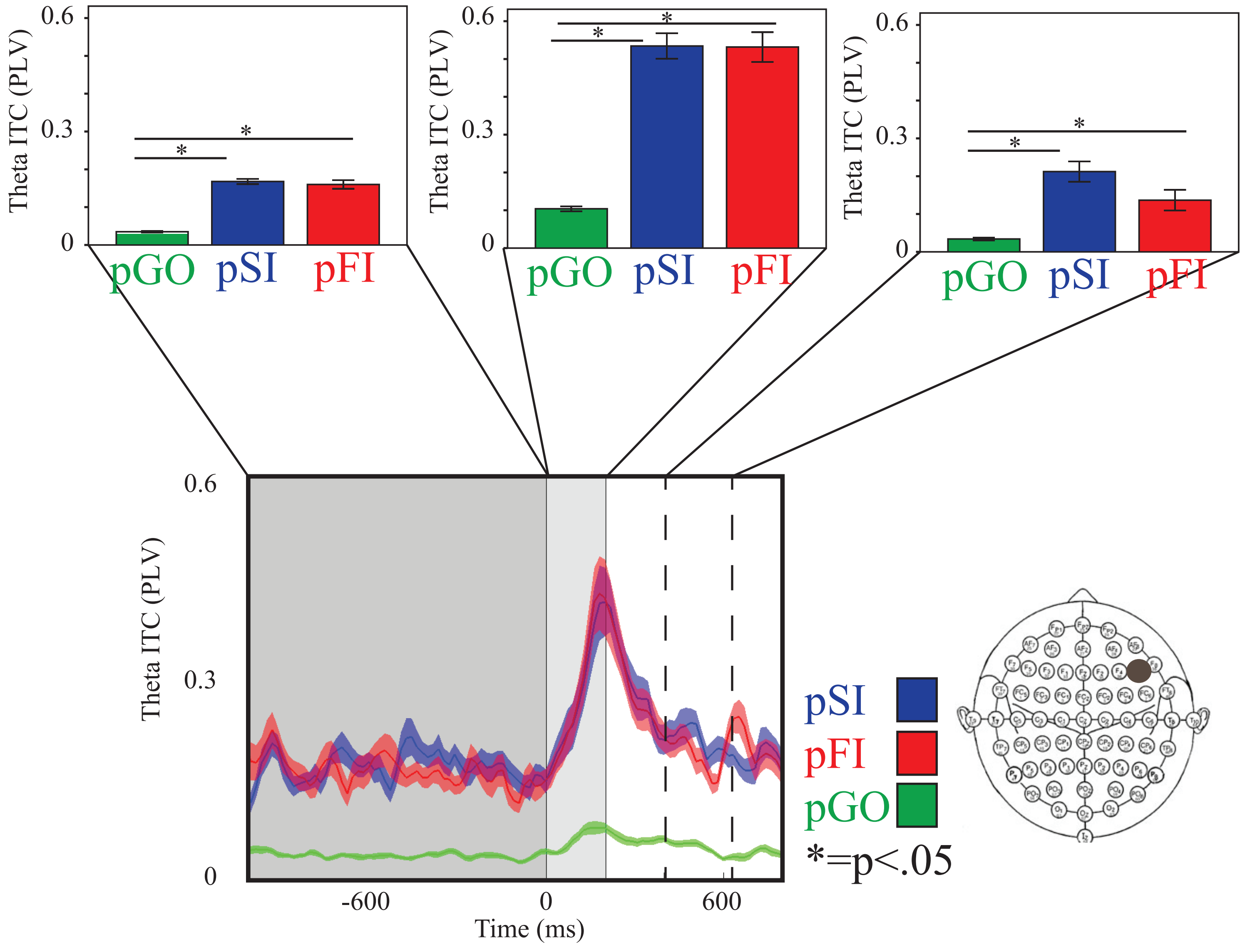

Supplementary Figure 4. Theta ITC at electrode F6. A) Bar graph displaying mean theta ITC averaged over the -1000 to 0ms interval, with 0ms as GO stimulus onset. B) Bar graph displaying the maximum ITC following GO stimulus onset (from 0 to 200ms). C) Theta ITC relative to the mean response time for each trial type.

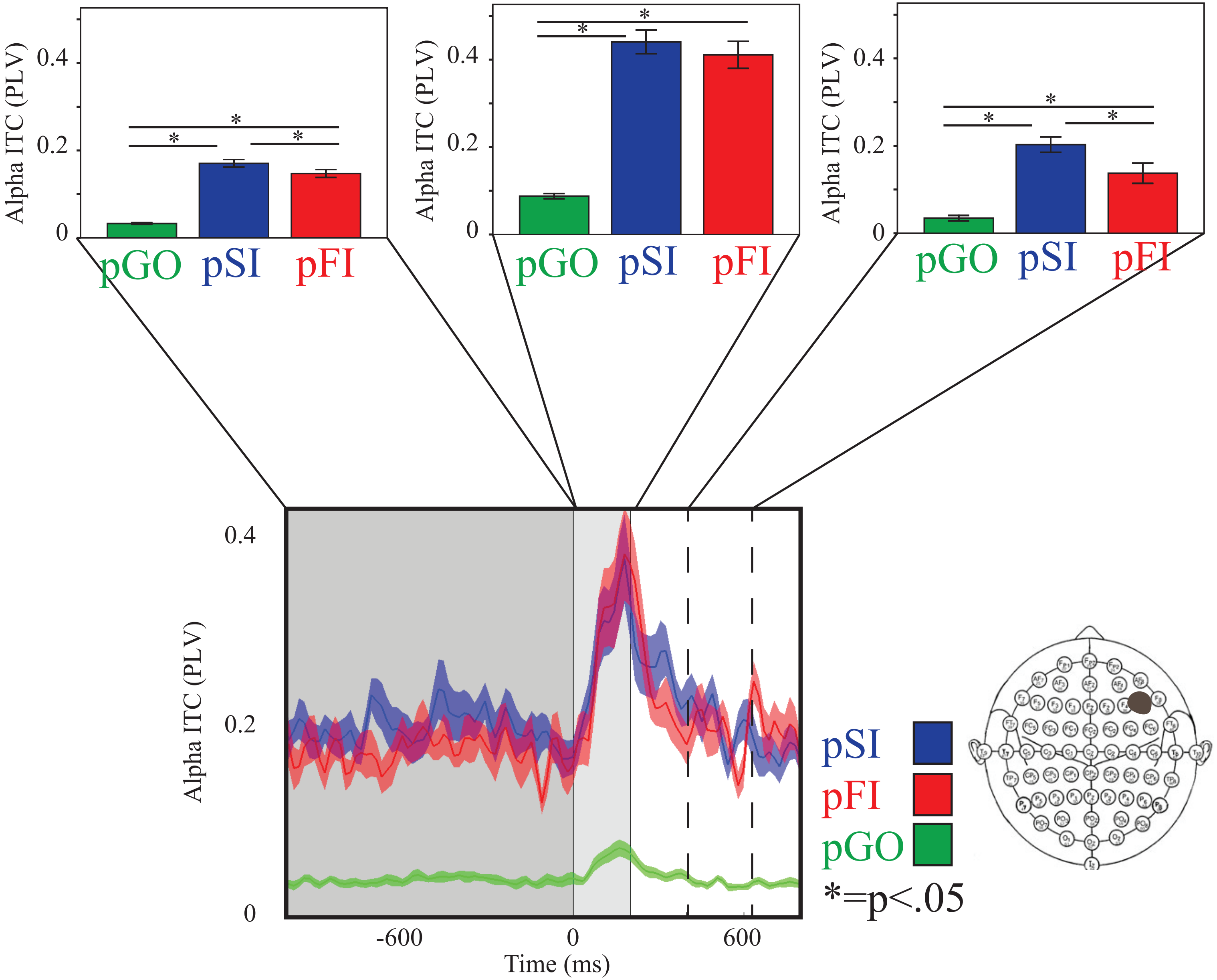

Supplementary Figure 5. Alpha ITC at electrode F6. A) Bar graph displaying mean alpha ITC averaged over the -1000 to 0ms interval, with 0ms as GO stimulus onset. B) Bar graph displaying the maximum ITC following GO stimulus onset (from 0 to 200ms). C) Alpha ITC relative to the mean response time for each trial type.

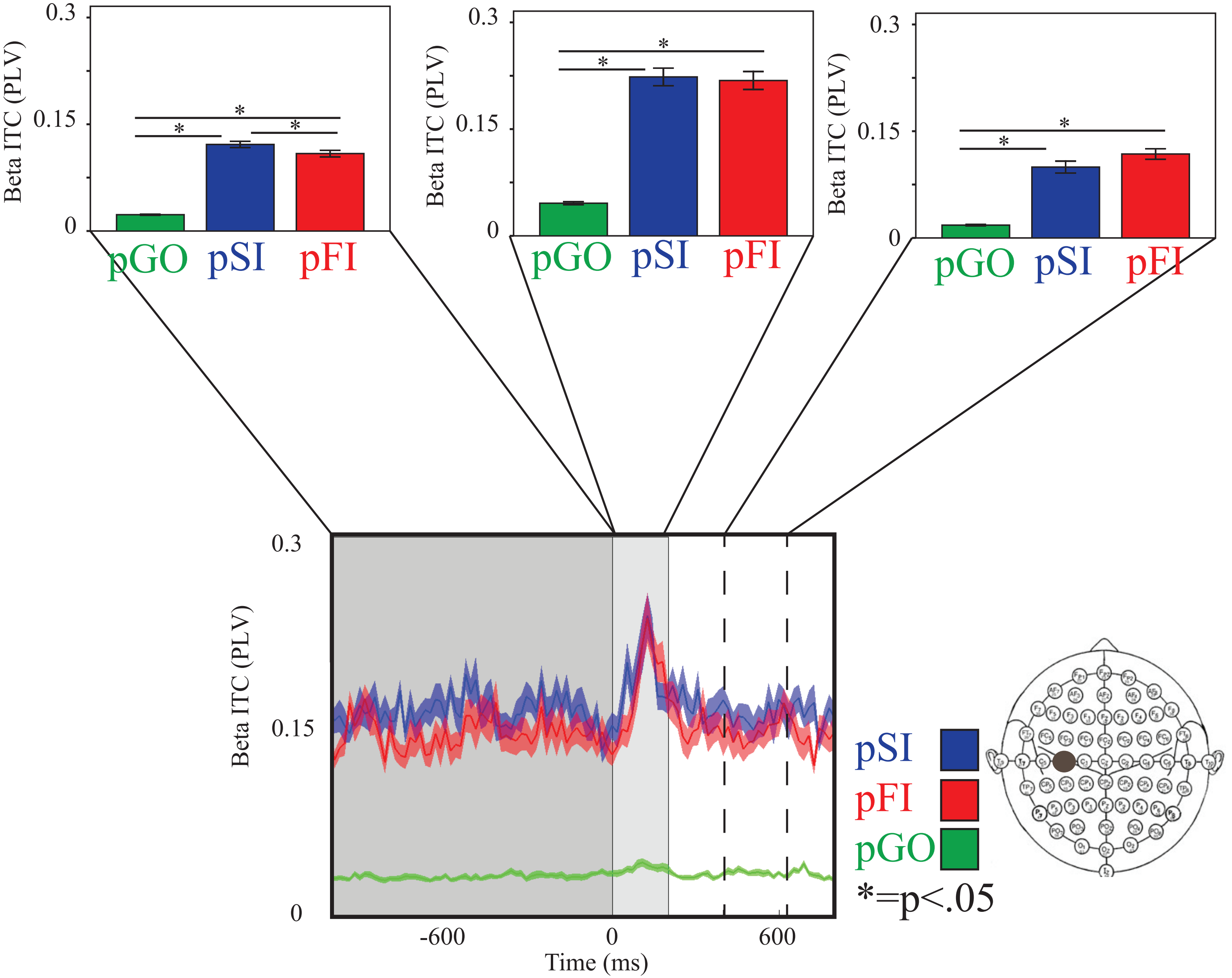

Supplementary Figure 6. Beta ITC at electrode C3. A) Bar graph displaying mean beta ITC averaged over the -1000 to 0ms interval, with 0ms as GO stimulus onset. B) Bar graph displaying the maximum ITC following GO stimulus onset (from 0 to 200ms). C) Beta ITC relative to the mean response time for each trial type.

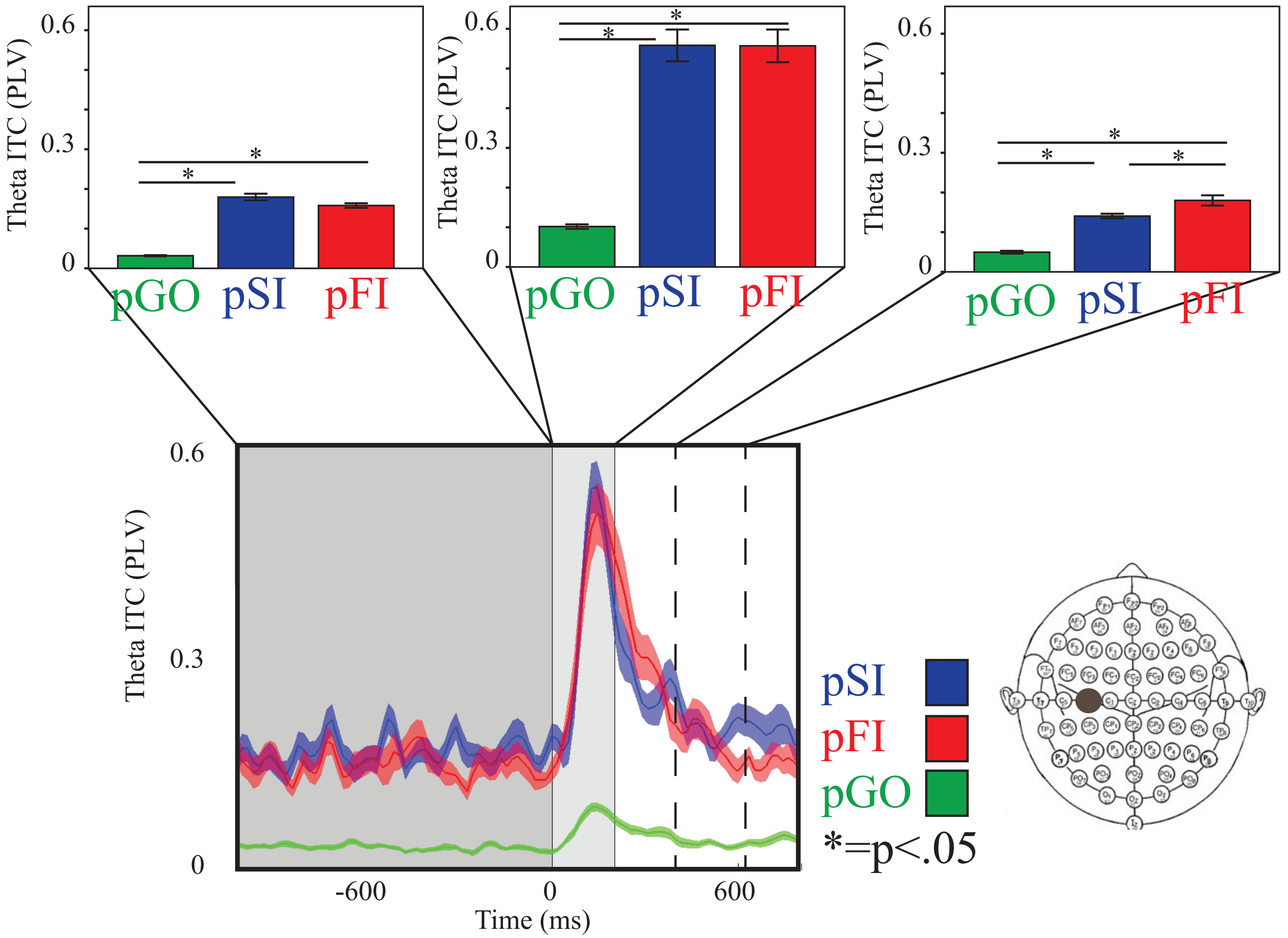

Supplementary Figure 7. Theta ITC at electrode C3. A) Bar graph displaying mean theta ITC averaged over the -1000 to 0ms interval, with 0ms as GO stimulus onset. B) Bar graph displaying the maximum ITC following GO stimulus onset (from 0 to 200ms). C) Theta ITC relative to the mean response time for each trial type.
